# Comparative Cytogenetics Analysis Among *Peckoltia* Species (Siluriformes, Loricariidae): Insights on Karyotype Evolution and Biogeography in the Amazon Region

**DOI:** 10.3389/fgene.2021.779464

**Published:** 2021-10-28

**Authors:** Kevin Santos da Silva, Augusto Cesar Paes de Souza, Ananda Marques Pety, Renata Coelho Rodrigues Noronha, Marcelo Ricardo Vicari, Julio Cesar Pieczarka, Cleusa Yoshiko Nagamachi

**Affiliations:** ^1^ Laboratório de Citogenética, Centro de Estudos Avançados da Biodiversidade, Instituto de Ciências Biológicas, Universidade Federal Do Pará, Belém, Brazil; ^2^ Laboratório de Estudos da Ictiofauna da Amazônia, Instituto Federal de Educação Ciência e Tecnologia Do Pará, Abaetetuba, Brazil; ^3^ Laboratório de Biologia Cromossômica, Estrutura e Função, Departamento de Biologia Estrutural, Molecular e Genética, Universidade Estadual de Ponta Grossa, Ponta Grossa, Brazil

**Keywords:** neotropical fish, snRNA, rDNA, biodiversity, amazon

## Abstract

*Peckoltia* is widely distributed genus in the Amazon and Orinoco basins and the Guiana Shield, containing 18 valid species, and distinct morphotypes still needing description in the scientific literature due to its great taxonomic complexity. This study performed a comparative chromosomal analysis of two undescribed *Peckoltia* species (*Peckoltia* sp. 3 Jarumã and *Peckoltia* sp. 4 Caripetuba) from the Brazilian Amazon using conventional chromosome bands methods and *in situ* localization of the repetitive DNA (*5S* and *18S rRNA* and *U1 snRNA* genes and telomeric sequences). Both species presented 2n = 52 but differed in their karyotype formula, probably due to inversions or translocations. The nucleolus organizer regions (NORs) showed distal location on a probably homeologous submetacentric pair in both species, besides an extra signal in a subtelocentric chromosome in *Peckoltia* sp. 4 Caripetuba. Heterochromatin occurred in large blocks, with different distributions in the species. The mapping of the 18S and 5S rDNA, and U1 snDNA showed differences in locations and number of sites. No interstitial telomeric sites were detected using the (TTAGGG)n probes. Despite 2n conservationism in *Peckoltia* species, the results showed variation in karyotype formulas, chromosomal bands, and locations of repetitive sites, demonstrating great chromosomal diversity. A proposal for *Peckoltia* karyotype evolution was inferred in this study based on the diversity of location and number of chromosomal markers analyzed. A comparative analysis with other *Peckoltia* karyotypes described in the literature, their biogeography patterns, and molecular phylogeny led to the hypothesis that the derived karyotype was raised in the left bank of the Amazon River.

## Introduction

The Loricariidae is one of the most specious family of catfish within the order Siluriformes, containing 1,016 valid species (Fricke et al., 2021). They are endemic to the Neotropical region, distributed throughout South America and part of Central America, and occur in a great diversity of habitats (Armbruster, 2004; Armbruster, 2008; Armbruster and Lujan, 2016). Analyzes based on morphological and molecular data support the recognition of six subfamilies: Lithogeninae, Delturinae, Hypoptopomatinae, Neoplecostominae, Loricariinae, and Hypostominae grouped in the tribes: Corymbophanini, Rhinelepini, Hypostomini, Pterygoplichthyini and Ancistrini (Armbruster, 2004; Lujan et al., 2015).

*Peckoltia* Miranda Ribeiro, 1912 (*sensu* Lujan et al., 2015), comprises 18 valid species, in addition to distinct morphotypes that still lack description in the scientific literature. They are widely distributed in the Amazon and Orinoco basins and the Guiana Shield (Armbruster et al., 2015; Lujan et al., 2015; Armbruster and Lujan, 2016). According to phylogenetic analyzes proposed for Loricariidae, *Peckoltia* genus receive strong support as monophyletic lineage (Lujan et al., 2015; Lujan et al., 2017; Roxo et al., 2019); however, a complex taxonomic identification procedure at a specific level related to a wide geographic distribution and morphological similarity is observed for these species (Armbruster et al., 2015; Armbruster and Lujan, 2016). For example, representatives of *Peckoltia vittate* (the type species of the genus) collected in the Amazon region (Xingu, Madeira and Orinoco rivers) presented polyphyletic lineages in molecular phylogeny by Lujan et al. (2015). Armbruster and Lujan (2016), analyzed morphological and molecular characters of samples collected in the Orinoco basin associated with *P. vittate* by Lujan et al. (2015) and described a new species, *Peckoltia wernekei*. These recent analyses agree that the diversity of *Peckoltia* species can be underestimated for the Amazon region.

Cytogenetic markers are important tools to analyze fish species possessing complex taxonomy (Bertollo et al., 2000; Centofante et al., 2003; Mariotto and Miyazawa, 2006) or to understand evolutionary features in groups with highly rearranged karyotypes (Nagamachi et al., 2010; Deon et al., 2020). Cytogenetic data are available for eight lineages of the genus *Peckoltia,* including valid species and unidentified morphotypes, collected at different points in the Amazon region. Despite all *Peckoltia* species share 2n = 52 chromosomes, variations in chromosome morphology, the number and position of NORs, distribution of the constitutive heterochromatin (CH) regions, and the presence of B chromosomes are observed among species of this genus (Table 1). Therefore, in *Peckoltia* species, many unique karyotypic features are observed that can be useful in recognizing distinct taxonomic units.

**TABLE 1:** Chromosomal diversity available in the literature and obtained in the present study for *Peckoltia* genus.

Species	Classic cytogenetics			Molecular cytogenetics				Loc.	Ref.
	2n	KF	Nor	18S rDNA	5S rDNA	U1 snDNA	Tel.		
*Peckoltia* sp*.* 1	52+1B	44m/sm+6st+2a+1B	multiple	-	-	-	-	A	1
*Peckoltia* sp*.* 2	52	32m/sm+18st+2a	multiple	-	-	-	-	A	1
*P. vittata*	52	36m/sm+14st+2a	simple	-	-	-	-	B	1
*P. vittata*	52	34m/sm+18st	simple	simple	multiple	-	-	B	2
*P. sabaji*	52	38m/sm+14st	multiple	multiple	multiple	-	-	B	2
*P. oligospila*	52	38m/sm+14st	multiple	multiple	simple	-	-	C	2
*P. cavatica*	52	38m/sm+14st	simple	multiple	multiple	-	-	C	2
*P. multipinis*	52	28m/sm+24st	simple	Simple	simple	-	-	B	2
*Peckoltia* sp. 3 Jarumã	52	46m/sm+6st	simple	simple	multiple	simple	distal	D	3
*Peckoltia* sp*.* 4 Caripetuba	52	40m/sm+12st	multiple	multiple	simple	multiple	distal	D	3

Locality: A—Monte Dourado, Pará state, Brazil; B—Altamira, Pará state, Brazil; C—Ourém, Pará state, Brazil; D—Abaetetuba, Pará state, Brazil. References: 1—de Souza et al., 2009; 2—Pety et al., 2018; 3—Present Study. Abbreviations: Diploid number (2n); Karyotype Formula (KF); Nucleolus Organizing Regions (NOR); Ribosomal RNA gene (rDNA); Small nuclear RNA gene (snDNA); Telomere (tel.); Supernumerary ou B chromosome (B); long arm (q); short arm (p); metacentric (m), submetacentric (sm), subtelocentric (st), acrocentric (a); Locality (Loc.); References (Ref.); (−) Data no available.

Repetitive DNAs are found in most eukaryotic genomes, representing important markers for molecular diversity analysis at the chromosomal level; they are organized in blocks (e.g., satellites and multigene families) or dispersed (e.g., transposons and retrotransposons). The contribution of the repetitive DNA for fish genome evolution has been evidenced (Vicari et al., 2010; Schemberger et al., 2019). The eukaryotic ribosomal DNA (rDNA) represents two multigene families with an organization *in tandem*: *45S ribosomal RNA* (*18S* + *5.8S* + *28S* genes) and *5S ribosomal RNA* (Long and Dawin, 1980). These genes are widely used in chromosomal studies in several organisms, including *Peckoltia* species (Pety et al., 2018), showing great molecular chromosomal diversity involving these sequences*.*

Small nuclear *RNA* genes (snDNA) represent another multigene family involved in the splicing and maturation process of messenger RNA encoded by the *U1*, *U2*, *U4*, *U5* and *U6 snRNA* genes (Busch et al., 1982). The snDNA sequences have been used as chromosomal markers for detailed comparative chromosome analysis in several groups of organisms, including fish, reptiles and arthropods (Cabral-de-Melo et al., 2012; Almeida et al., 2017; Cavalcante et al., 2020; Dulz et al., 2020).

In this study, we describe the karyotypes of two undescribed *Peckoltia* species, first time sampled in the Tocantins River basin in Brazil, and compare them with cytogenetic data available in the literature. From this, we discuss the possible mechanisms of karyotypic diversification, biogeography and their evolutionary implications for this genus.

## Materials and Methods

### Samples

**FIGURE 1:**
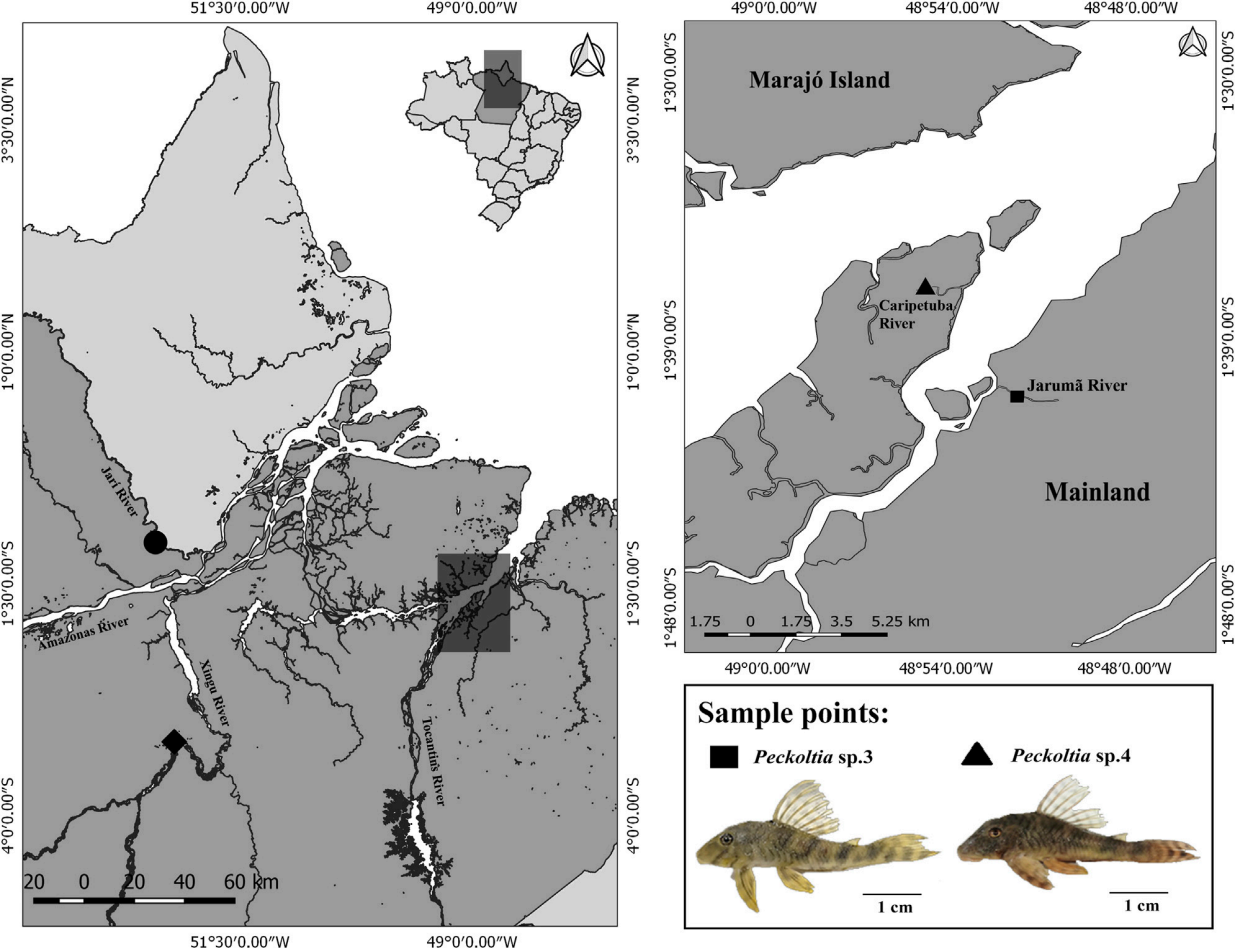
Geographical location of sampling sites for *Peckoltia* sp. 3 Jarumã (square) and *Peckoltia* sp. 4 Caripetuba (triangle) in the present study. The coordinates of the sampling sites for *Peckoltia vittata* (diamond), *Peckoltia* sp. 1, and *Peckoltia* sp. 2 (circle) reported by de Souza et al. (2009) are also shown. The map was made using QUANTUM-GIS (Q-GIS) v. 3.4.5. The database was obtained from Instituto Brasileiro de Geografia e Estatística—IBGE. An *Peckoltia* specimen is shown below. Scale bar: 1 cm. Photo by KSS.

Samples of the two morphologically different but still undescribed species of *Peckoltia* named *Peckoltia* sp. 3 Jarumã and *Peckoltia* sp. 4 Caripetuba, after the rivers they were collected in different hydrographic points in the Tocantins River basins of northern Brazil were analyzed (Figure 1). The taxonomic identification of the sample was checked using the identification key proposed by Armbruster and Lujan (2016), Armbruster (2008). The results show that the specimens do not fit into any of the species already Figure 1described. The collection points, number of individuals, sex, and voucher of deposits in the zoological collection are shown in Table 2. The samples were obtained under a permanent field permit obtained by JCP (number 13248 from “Instituto Chico Mendes de Conservação da Biodiversidade”). The Cytogenetics Laboratory from UFPA has permit number 19/2003 from the Ministry of Environment for sample transport and permit 52/2003 to use the samples for research. The Ethics Committee (Comitê de Ética Animal da Universidade Federal do Pará) approved this research (Permit 68/2015). The specimens have been deposited in the ichthyological collection of the Museu Paraense Emílio Goeldii (MPEG) (Belém, Brazil).

**TABLE 2:** Samples and collection sites for *Peckoltia* species analyzed in this study.

Species	Sex		River	City/State	Voucher	Coordinates	
*Peckoltia* sp. 3	2♂	4♀	Jarumã River	Abaetetuba-PA	MPEG 38949	01°42′41.9″S	48°51′45.9″W
*Peckoltia* sp. 4	1♂	1♀	Caripetuba River	Abaetetuba-PA	MPEG 38950	01°37′23.49″S	48°55′33″W

MPEG—Museu Paraense Emilio Goeldi.

### Chromosomal Analysis

Mitotic chromosomes were obtained from kidney cells after *in vivo* colchicine treatment as described (Bertollo et al., 1978). The animals were anesthetized with eugenol and subsequently sacrificed for the removal of kidney cells. Metaphases were analyzed by conventional Giemsa, C-banding (Sumner, 1972) and AgNO_3_ staining (Howell and Black, 1980). Fluorescence *in situ* hybridization (FISH) was undertaken as described (Martins and Galetti, 1999) using a general telomere probe for vertebrates, 18S rDNA, 5S rDNA, and U1 snDNA probes.

### Probes Labeling and *in situ* Localization

DNA extraction was performed using PureLink Genomic DNA Mini Kit (Invitrogen) following the manufacturer’s instructions. The probes were obtained from a PCR using genomic DNA of *Peckoltia* sp. 3 Jarumã and *Peckoltia* sp. 4 Caripetuba with primers previously described for 18S rDNA (Hatanaka and Galetti Jr, 2004), for 5S rDNA (Suarez et al., 2017) and U1 snDNA (Cabral-de Melo et al., 2012). These probes were labeled by nick-translation with biotin or digoxigenin. Telomeric probes were obtained from PCR using the set of primers F-5′(TTAGGG)_5_-3′ and R-5′(CCCTAA)_5_-3′ followed by labeling with Digoxigenin-11-dUTP (Roche Applied Science^®^) (Ijdo et al., 1991). Fluorescence *in situ* hybridization (FISH) was performed as described by Martins and Galetti (1999) using the following stringency conditions: 2.5 ng/μL of each probe, 50% formamide, 2 x SSC, 10% dextran sulfate, and hybridization at 42°C for 16 h. Fluorescent signals were detected using Streptavidin Alexa Fluor 488 (Molecular Probes, Carlsbad, CA, United States) and anti-digoxigenin rhodamine Fab fragments (Roche Applied Science, Penzberg, Germany). Chromosomes were counterstained with 0.2 μg/ml of 4′6-diamidino-2-phenylindole (DAPI) in Vectashield mounting medium (Vector, Burlingame, CA, United States).

### Microscopic Analysis and Image Capture

At least 30 metaphases Giemsa-stained per individual were analyzed for confirming the diploid number, karyotypic structure, and chromosomal markers. Cytogenetic images of Giemsa-stained chromosomes were obtained using an Olympus BX41 microscope (bright field/phase) with a digital camera CCD 1300QDS and analyzed using GenASIs software version 7.2.7.34276 from ASI (Applied Spectral Imaging). FISH images were obtained using a Nikon H550S microscope and analyzed using the Nis-Elements software. Images were adjusted using Adobe Photoshop CS6 software. Chromosomal morphology was classified according to literature (Levan et al., 1964), with adaptations.

## Results

**FIGURE 2:**
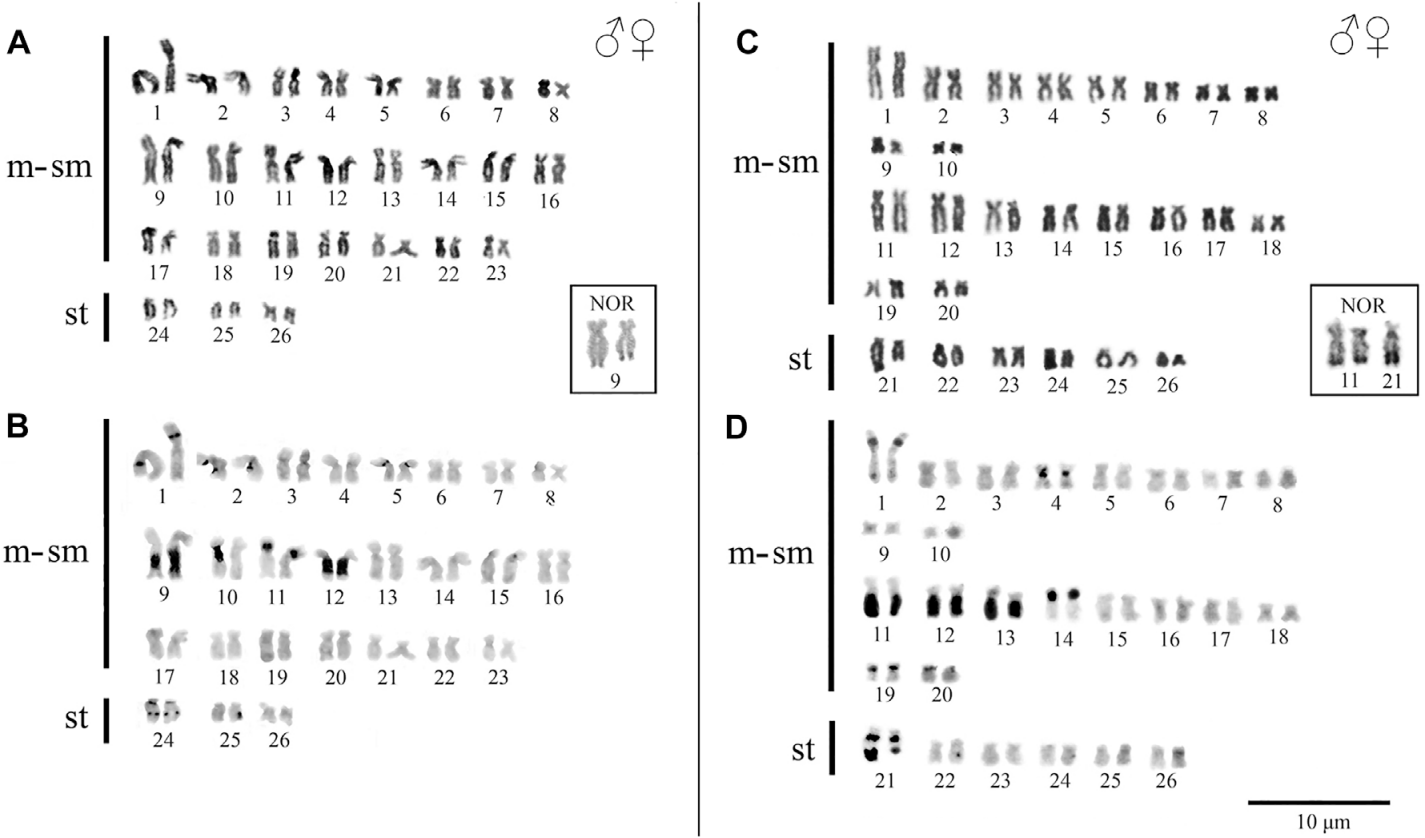
Karyotypes of *Peckoltia* species. In **(A)** conventional staining and **(B)** C-banding of *Peckoltia* sp. 3 Jarumã; in **(C)** conventional staining and **(D)** C-banding of *Peckoltia* sp. 4 Caripetuba. In box the Nucleolus Organizing Regions (NORs).

*Peckoltia* sp. 3 Jarumã and *Peckoltia* sp. 4 Caripetuba species presented 2n = 52 chromosomes and karyotype formulas (KF) 46m/sm + 6st, and 40m/sm + 12st, respectively. Heteromorphic sex chromosomes were not identified in the karyotypes described here (Figures 2A,C).

In *Peckoltia* sp. 3 Jarumã, heterochromatin occurred in the interstitial region of the short arm (p) of pairs 1p, 5p and 11p; in large blocks in the long arm (q) in pairs 9q with size heteromorphism and 12q; and in the pericentromeric and interstitial region of the 24q pair (Figure 2B). In *Peckoltia* sp. 4 Caripetuba, heterochromatin occurred in the interstitial region of pairs 1p, 14p, 19p and 20p; in the centromeric region of par 4; in large blocks in pairs 11q, 12q and 13q and in the pericentromeric and distal region of pair 21q, in which there is a marked heteromorphism in size between the homologues (Figure 2D). The Ag-NORs were located distal at pair 9q adjacent to the heterochromatin block in *Peckoltia* sp. 3 Jarumã (Figure 2 in box), and at pair 11q, coincident with heterochromatin and in one of the homologues in pair 21q in *Peckoltia* sp. 4 Caripetuba (Figure 2 in box).

**FIGURE 3:**
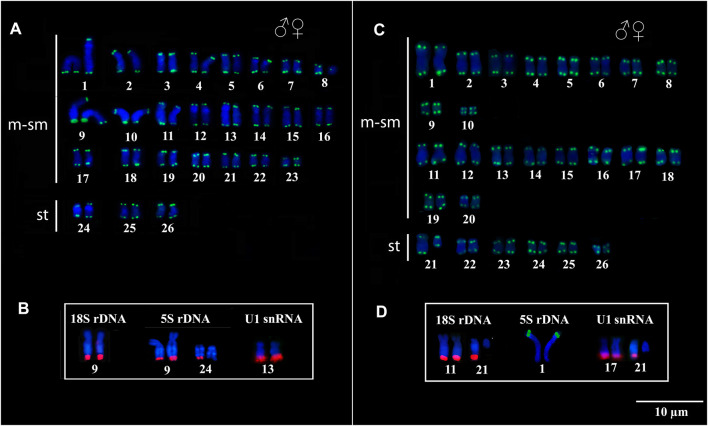
Fluorescent *in situ* Hybridization indicating the physical location of the Telomeric probes, 18S rDNA, 5S rDNA and U1 snDNA probes in *Peckoltia* species. In *Peckoltia* sp. 3 Jarumã: **(A)** Telomeric sequence (green) and **(B)** 18S rDNA (red), 5S rDNA (red) and U1 snDNA (red). In *Peckoltia* sp. 4 Caripetuba: (**C**) Telomeric sequence (green) and **(D)** 18S rDNA (red), 5S rDNA (green) and U1 snDNA (red).

Telomeric sequences occurred in the distal region of all chromosomes in both species, with no evidence of ITS vestiges (Figures 3A,C). The 18S rDNA, 5S rDNA, and U1 snDNA probes hybridized at the distal position of the chromosomes in the karyotypes of both species. In *Peckoltia* sp. 3 Jarumã, both 18S and 5S rDNA are colocalized in the 9q pair, in addition to a 5S rDNA site in one of the homologues in the 24q pair, and U1 snDNA is located in the distal region of the pair 13q (Figure 3B). In *Peckoltia* sp. 4 Caripetuba, 18S rDNA is located in pair 11q, in an additional site in one homologue of pair 21q; 5S rDNA is located in pair 1p; and U1 snDNA is located in the distal position of the pair 17q, with an additionnal site in one homologue of pair 21q colocalized with heterochromatin and 18S rDNA site (Figure 3D).

## Discussion

### Karyotype Diversity in *Peckoltia*

Cytogenetic information for the species of *Peckoltia* is described in Table 1 and, despite the occurrence of 2n = 52 chromosomes, all species of *Peckoltia* show different KF. These differences can be explained by inversions or translocations, which represent important mechanisms of karyotypic diversification in Loricariidae (Kavalco et al., 2005; Mariotto et al., 2011). Alternatively, centromeric repositioning has also been proposed to cause variations in chromosome morphology with no change in diploid number (Montefalcone et al., 1999; Rocchi et al., 2012). However, the occurrence of this mechanism in Loricariidae karyotypes still needs deep investigation.

The Loricariidae has extensive chromosomal diversity, with variation in diploid number from 34 to 96 chromosomes, with a putative ancestral karyotype showing 2n = 54 chromosomes (Artoni and Bertollo, 2001). The reduction to 2n = 52 was probably due to a Robertsonian fusion in an ancient common ancestor in Ancistrini representatives with no ITS manutention (Bueno et al., 2018).

In Loricariidae, it is expected the presence of heterochromatic regions distributed in blocks on few chromosomes (Ziemniczak et al., 2012). Interestingly, in *Peckoltia*, extensive heterochromatic blocks are observed, some occupying a large part of the long arms of submetacentric/subtelocentric chromosomes (de Souza et al., 2009, present study). The presence of large heterochromatic blocks on morphologically similar chromosomes may suggest a shared character in *Peckoltia* karyotypes, as proposed previously (de Souza et al., 2009). However, it is known that heterochromatic regions are characterized by great diversity in highly repetitive DNA content (Dimitri et al., 2009) and may not reflect chromosomal homologies in *Peckoltia* species, as visualized among the species analyzed here by FISH with repetitive sequences. Noteworthy, the distribution of heterochromatin, and different repetitive sequences, observed in pairs 24 in *Peckoltia* sp. 3 Jarumã and 21 in *Peckoltia* sp. 4 Caripetuba, makes these chromosomes good cytotaxonomic markers, and both represent unique characteristics in each of these species.

Other plesiomorphic conditions for Loricariidae include the 18S and 5S rDNA sequences in a single pair of meta/submetacentric chromosomes (Ziemniczak et al., 2012). However, in the Ancistrini tribe, both the synteny and non-synteny between the 18S and 5S sequences are commonly observed (Mariotto et al., 2011; Ribeiro et al., 2015; Favarato et al., 2016; Prizon et al., 2016; Pety et al., 2018), showing the huge chromosome sites variation in the karyotypes of this group (Pansonato-Alves et al., 2013; Bueno et al., 2014; Pety et al., 2018). The mapping of 18S and the 5S sequences in the karyotypes here described is in agreement with that observed in other species of *Peckoltia*; in which extensive dispersion of these genes is observed (Pety et al., 2018) (Table 1). This dispersion of rDNA in the genomes of Loricariidae can be explained either by the association of these genes with other repetitive sequences, including transposable elements or by the evolutionary breakpoint regions close to rDNA sites promoting chromosome rearrangements (Barros et al., 2017; Glugoski et al., 2018, 2020; Deon et al., 2020). Furthermore, the heterochromatic condition involving clusters of rDNA suggests that other repetitive DNA classes, around 45S and 5S rDNA sequences, may promote their chromosomal dispersion in the *Peckoltia* species analyzed here, as shown for other species of Loricariidae (Glugoski et al., 2018; Deon et al., 2020).

In fish, the *snRNA* genes have shown great diversity of the pattern of chromosomal localization (Úbeda-Manzanaro et al., 2010; Cabral-de-Melo et al., 2012; Scacchetti et al., 2015; Yano et al., 2017). In this work, the U1 snDNA sequence was mapped for the first time in Loricariidae species, showing location in a pair of submetacentric chromosomes in both species, in addition to an extra site in one of the homologues of pair 21 of *Peckoltia* sp. 4 Caripetuba (Figure 3D). The snDNA sequences are considerably more stable at the chromosomal level when compared to rDNA (Cabral-de-Melo et al., 2012). However, we observed that among the karyotypes of *Peckoltia* analyzed here, the U1 snDNA probes show variation in the number of chromosomal sites similar to that observed for the rDNA (Table 1). These data indicate that these gene families can be equally dynamic in the genomes of species of *Peckoltia.* Several chromosomal sites of rDNA and snDNA sequences are observed in different groups of fish, such as species of the Loricariidae, Cichlidae and Anostomidae families; the emergence of new chromosomal sites is related to the association of these sequences with active mobile elements in these organisms (Kapitonov and Jurka, 2006; Cabral-de-Melo et al., 2012; Dulz et al., 2020). Future analyzes of rDNA and snDNA nucleotide sequences will be essential to verify the possible involvement of transposable elements in the movement of these sequences in the genomes of *Peckoltia* species.

### Biogeography Hypothesis in *Peckoltia*

The putative ancestral karyotype for Loricariidae has 2n = 54, a single NOR and gene synteny for 5S and 18S rDNA sequences (Ziemniczak et al., 2012). The tribes belonging to the Hypostominae subfamily share a common ancestor (Armbruster, 2004; Lujan et al., 2015) that possibly had a 2n = 52 chromosomes (Bueno et al., 2018). Thus, variations in the 2n, multiple NOR and synteny break between 5S and 18S would represent derived characteristics that can be apomorphic or homoplasic. Analyses involving 18S and 5S rDNA and U1 snDNA show the importance of these sequences as markers of karyotype diversification in the *Peckoltia* genus.

**FIGURE 4:**
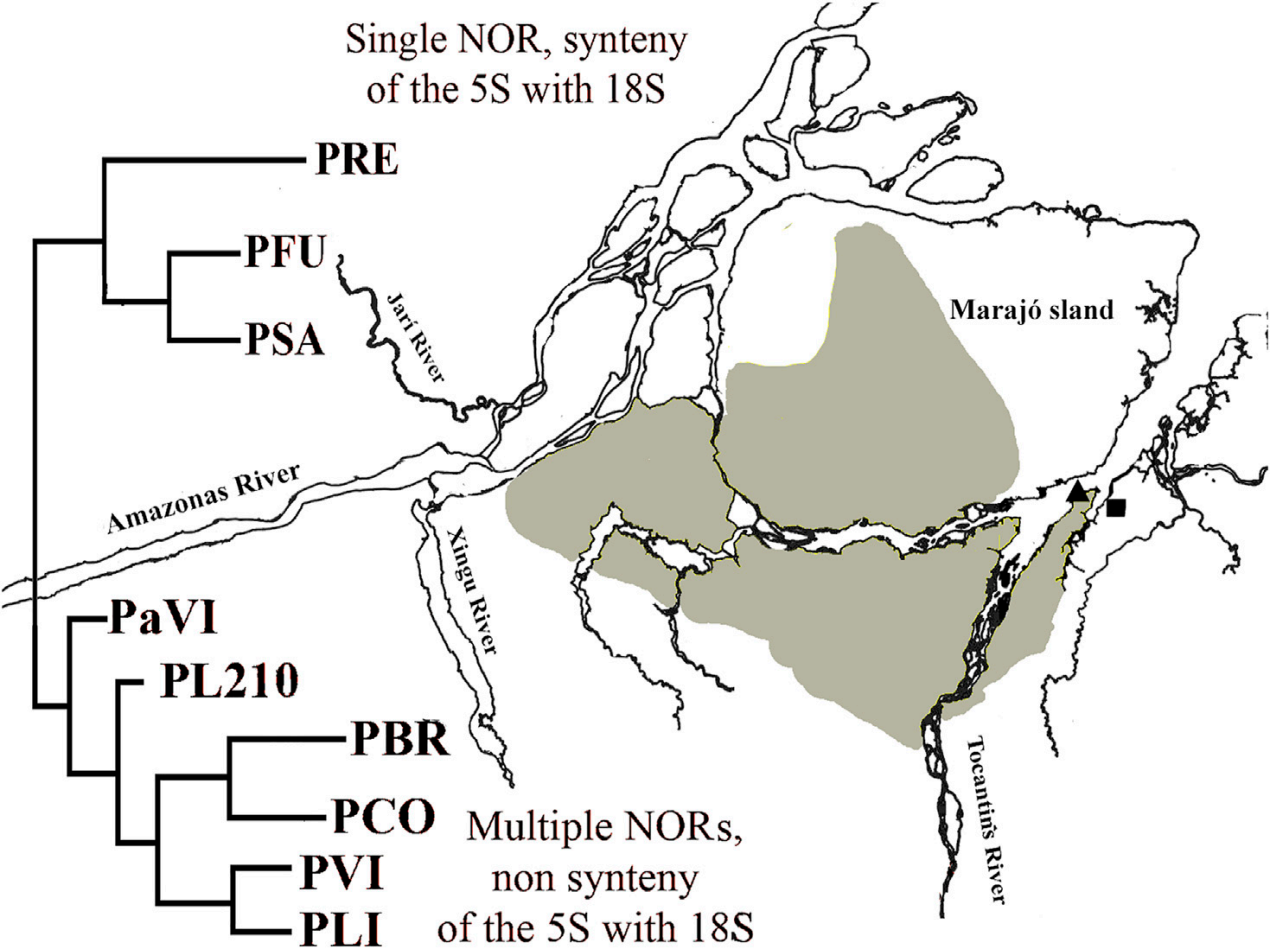
Map of the mouth of Amazon River with a simplified version of *Peckoltia* phylogeny (in red) by Lujan et al. (2017). PRE: *Peckoltia relictum*; PFU: *Peckoltia furcatum*; PSA: *Peckoltia sabaji*; PaVI: *Peckoltia* aff. *vittata*; PL210: *Peckoltia* n. sp. L210; PBR: *Peckoltia braueri*; PCO: *Peckoltia compta*; PVI: *Peckoltia vittata*; PLI: *Peckoltia lineola*. Geographical location of sampling sites for *Peckoltia* sp. 3 Jarumã (square) and *Peckoltia* sp. 4 Caripetuba (triangle) in the present study. Yellow: the distribution of the Plio-Pleistocene/Pleistocene Post-Barreiras sediments (Rossetti and Valeriano, 2007).

Phylogenetic analyzes support the monophyly of the *Peckoltia* genus (Lujan et al., 2015, 2017; Roxo et al., 2019). A phylogeny for the *Peckoltia* clade proposed by Lujan et al. (2017), based on the concatenated sequences of two mitochondrial genes (16S, Cyt b) and three nuclear genes (RAG1, RAG2, MyH6), has two well-defined branches, which are sister groups and two branches with non-defined relationships. One of the defined branches presents *P. vittata* (single NOR, synteny of the 5S with 18S) (Pety et al., 2017), *P. compta*, *P. braueri* and *P. lineola* (karyotypes not described); and the other branch with *P. sabaji* (multiple NORs, non-synteny of the 5S with 18S) (Pety et al., 2018), *P. furcata* and *P. relictum* (karyotypes not described). Noteworthy, most of the specimens from the branch with *P. vittata* are on the right bank of the lower Amazon River, and those from the branch with *P. sabaji* are on the left bank. The karyotype of *Peckoltia* sp. 4 Caripetuba would be more similar to that of *Peckoltia* sp. 1 and 2 previously described (de Souza et al., 2009), collected on the left bank of the Amazon River and with karyotypic characteristics derived from the ancestral karyotype proposed for Loricariidae. Thus, it is possible that these derived features, such as multiple rDNA and NORs sites, have arisen in this region (Figure 4).

In the present work, the karyotype of *Peckoltia* sp. 4 Caripetuba has several derived characteristics, being found on the right bank of the Amazon River. This fact can be explained if we consider the paleogeography of the region. The continental portion of Abaetetuba (Rio Jarumã) comprises the Barreiras Formation (Miocene). In turn, the Post-Barreiras Formation (Plio-Pleistocene) filled the paleovale of the Tocantins River, diverting this river (which originally crossed Marajó Island to its northern portion) and thus splitting Marajó Island from the mainland, originating Rio Pará and the islands in front of the city of Abaetetuba-PA (Figure 4), where the Caripetuba River is found (Rossetti and Valeriano, 2007). Therefore, the rivers where the karyotype of *Peckoltia* sp. 4 Caripetuba is found are in islands considerably newer than the one where *Peckoltia* sp. 3 Jarumã is found. These islands are covered by the Post-Barreiras Formation, being connected to the western portion of Marajó Island (Tatumi et al., 2007), not related with the continental region where the Jarumã River is located. The Marajó Island, in turn, is a communication corridor, connecting biodiversity on the left bank of the Amazon River with biodiversity on the right bank (Fernandes et al., 1995). This fact may explain why the most recent karyotype (*Peckoltia* sp. 4 Caripetuba) is located in this region. It will be important to test by molecular markers whether the *Peckoltia*-associated morphotypes analyzed in this study and by de Souza et al. (2009), which have derived karyotypes, belong to the *P. sabaji* branch.

This study describes the karyotypes of two undescribed species of *Peckoltia* and compares them with available chromosomal data for the genus. The maintenance of the same 2n in the species of *Peckoltia* may suggest that this genus has conserved karyotypes; however, the variations observed in the KF, NOR, heterochromatin and 18S and 5S rDNA sequences between the karyotypes of the species in this study compared to those previously described suggest great interspecific diversity in the genus. Furthermore, the differential localization of the U1 snDNA sequence among the karyotypes described here corroborates the involvement of repetitive sequences in the diversification of the genomes of these species. Therefore, due to the considerable cytogenetic diversity, species-specific characters were observed, showing great potential for identifying distinct taxonomic lineages in *Peckoltia*, in addition to demonstrating that the karyotypic variation in this genus is much greater than conventional staining suggests. Future analyzes considering the geographic distribution of *Peckoltia* species with primitive versus derived karyotypic characteristics compared to molecular phylogenies may provide relevant information about its evolutionary history.

## Data Availability

The original contributions presented in the study are included in the article/Supplementary Material, further inquiries can be directed to the corresponding author.
